# Deep learning-based localization algorithms on fluorescence human brain 3D reconstruction: a comparative study using stereology as a reference

**DOI:** 10.1038/s41598-024-65092-3

**Published:** 2024-06-25

**Authors:** Curzio Checcucci, Bridget Wicinski, Giacomo Mazzamuto, Marina Scardigli, Josephine Ramazzotti, Niamh Brady, Francesco S. Pavone, Patrick R. Hof, Irene Costantini, Paolo Frasconi

**Affiliations:** 1 Department of Information Engineering, University of Florence, 50100 Firenze, FI Italy; 2 Nash Family Department of Neuroscience, Friedman Brain Institute and Center for Discovery and Innovation, Icahn School of Medicine at Mount Sinai, New York, NY 10019 USA; 3 European Laboratory for Non-Linear Spectroscopy (LENS), 50019 Sesto Fiorentino, FI Italy; 4 National Research Council, National Institute of Optics (CNR-INO), 50019 Sesto Fiorentino, FI Italy; 5 Department of Experimental and Clinical Medicine, University of Florence, 50100 Firenze, FI Italy; 6 Department of Physics, University of Florence, 50019 Sesto Fiorentino, FI Italy; 7 Department of Biology, University of Florence, 50019 Sesto Fiorentino, FI Italy

**Keywords:** Cell detection, Deep-learning, Human brain, Broca’s area, 3D reconstruction, Fluorescence microscopy, Stereology, Bioinformatics, Microscopy, Biological fluorescence, Computer science, Computational models, Computational neuroscience, Computational platforms and environments, High-throughput screening, Image processing, Machine learning, Computational neuroscience, Computational biology and bioinformatics, Neuroscience

## Abstract

3D reconstruction of human brain volumes at high resolution is now possible thanks to advancements in tissue clearing methods and fluorescence microscopy techniques. Analyzing the massive data produced with these approaches requires automatic methods able to perform fast and accurate cell counting and localization. Recent advances in deep learning have enabled the development of various tools for cell segmentation. However, accurate quantification of neurons in the human brain presents specific challenges, such as high pixel intensity variability, autofluorescence, non-specific fluorescence and very large size of data. In this paper, we provide a thorough empirical evaluation of three techniques based on deep learning (StarDist, CellPose and BCFind-v2, an updated version of BCFind) using a recently introduced three-dimensional stereological design as a reference for large-scale insights. As a representative problem in human brain analysis, we focus on a $4~\text {-cm}^3$ portion of the Broca’s area. We aim at helping users in selecting appropriate techniques depending on their research objectives. To this end, we compare methods along various dimensions of analysis, including correctness of the predicted density and localization, computational efficiency, and human annotation effort. Our results suggest that deep learning approaches are very effective, have a high throughput providing each cell 3D location, and obtain results comparable to the estimates of the adopted stereological design.

## Introduction

The accurate quantification of neurons in specific brain regions is of utmost importance for understanding the intricate organization and function of the human brain. Unbiased stereology is a well-established method for obtaining a quantitative estimation of geometric properties in 3D images by sampling the volume with planar probes^1^ and is the current method of choice for counting brain cells^1,2^. However, with the rapid advancements in artificial intelligence (AI) and computer vision, novel approaches have emerged, offering biologists and neuroscientists the potential to not only estimate densities but also obtain spatial coordinates of individual neurons^3–6^, a much finer-grained estimation of neuron densities. Supervised segmentation algorithms based on deep learning (DL) can learn from relatively small volumes manually annotated with somata coordinates and predict the somata locations in new (unseen) volumes. 2D deep learning techniques have been employed in conjunction with unbiased stereology^7^ and have been reported to produce count estimates that highly correlate with classic stereological approaches^8^. A related AI-based approach^9^ has been shown to reduce the error rate of unbiased stereology estimates on novel test images; it also operates in 2D and requires a human-in-the-loop procedure. Importantly, AI techniques can also be directly applied to whole 3D images^3,4^, which can help to disambiguate cells from various sources of noise since the model has access to a much wider spatial context.

Advancements in tissue clearing methods in combination with fluorescence microscopy techniques, in particular light-sheet fluorescence microscopy (LSFM), have spread the possibility of performing cm-sized volumetric reconstruction of biological specimens, such as the human brain^10–14^. However, the development of automatic tools able to perform cell counting on human brain 3D reconstruction is still in its early stage. Human brain data present specific challenges that need to be solved to obtain an efficient and applicable cell-counting method. The imaging data present tremendous variation through the same sample in terms of contrast homogeneity and specificity of signals to be recognized. Moreover, compared to the mouse brain, the human brain presents high autofluorescence signals coming from the prolonged fixation of the tissue and the non-specific fluorescence emitted by endogenous pigments such as lipofuscin in aged neurons and in erythrocytes from the retained inside the blood vessels. And, even if tissue preparation precautions can mitigate the inhomogeneity of the signals through the sample (see Sect. "Human brain fluorescence imaging" for details), classical post-processing methods (e.g., modification of image contrast) cannot be applied to the whole analyzed volume due to the variability of biological samples. The large size of the human brain ($1400~\text { cm}^3$) compared to the mouse brain ($1\text { cm}^3$) also leads to the production of a massive quantity of data that needs to be analyzed on a time-scale comparable at least to that of imaging.

In this study, we present a comprehensive comparative analysis of three AI-based methods (CellPose^5^, StarDist^4^, and an updated version of BCFind^3^, from now on referred as BCFind-v2) using a new stereologic design^12^, explicitly developed for thick ($\sim 450$ $\mu$m) cleared sections of human brain 3D reconstructions acquired with LSFM, as a reference for large-scale evaluation. Unlike previous studies, we encompass multiple dimensions of analysis by comparing predicted cell localization (on small, annotated volumes) and density (on whole brain slabs), runtime, and manual annotation efforts, to facilitate a better informed decision in selecting the most effective approach. We report our analyses on a large-scale 3D data set derived from nine scans of a human Broca’s area, representing an approximate volume of $3.2~\text { cm}^3$. Models were trained and evaluated on 54 volumes (22 596 annotated cells) using a leave-one-slab-out procedure to ensure that predictions were evaluated on slabs not used for training. Four slabs were entirely analyzed, obtaining soma coordinates of all present neurons for a total of $\sim 12$ million detected cells.

Broca’s area (Brodmann’s area 44/45) is a neocortical region characterized by a distinct cytoarchitecture^15,16^ , well-established neural connectivity^17–19^, remarkable adaptability^20,21^ and plays a central role in language production and comprehension^22–24^, but its precise functions, individual variability, and the effects of damage remain areas of active investigation^18,25,26^. An accurate and detailed localization of neuron types in this area has the potential of providing novel insights on its function, cellular specialization, and connectivity.

## Results

To assess the reliability of DL-based methods and their applicability to cell counting in large-scale 3D LSFM brain reconstructions, we focus on diverse aspects that characterize the prediction quality, both at a granular level (comparing annotated and DL-predicted cellular coordinates) and at a cortical layer level (comparing densities obtained by unbiased stereology against those obtained from DL methods). Because DL methods are computationally demanding, we also compare their speed at inference time. We finally compare the human annotation effort for DL methods and for unbiased stereology. Human brain data is acquired by cutting the whole Broca’s area tissue into slices or slabs (see Sect. "Human brain fluorescence imaging" for details). These two terms will be used interchangeably throughout the text. All results of this work have been obtained from nine slabs of the Broca’s area from a single human subject: DL models have been trained and evaluated on 54 random volumes taken from slabs 1–6, while cell locations through the whole acquired volume were obtained on slabs 6, 18, 30 and 42 and compared to stereology estimates and annotations.

### Cell localization

**Figure 1:**
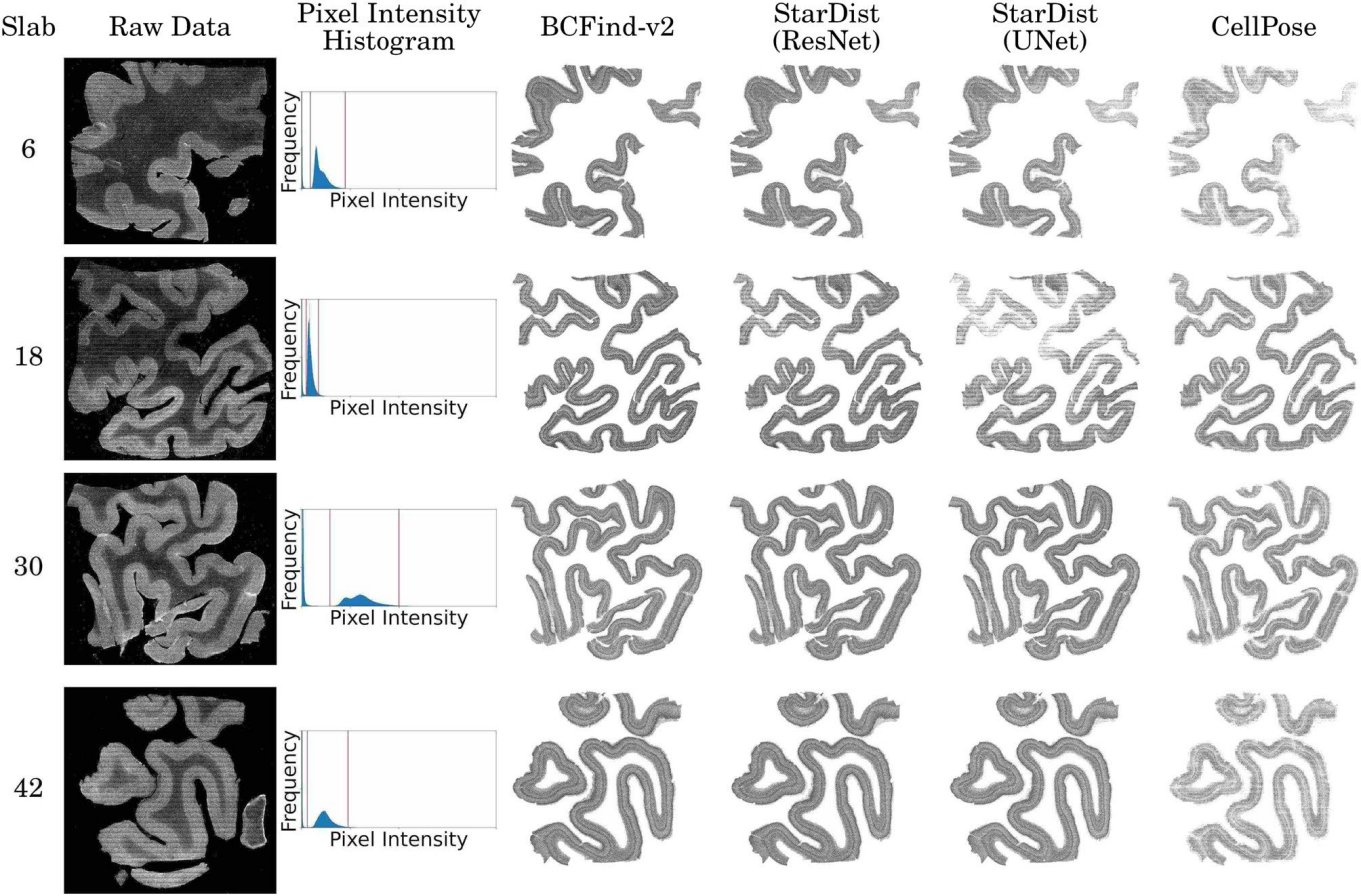
Maximum intensity projections (MIP) of four brain slabs from the considered human Broca’s area, corresponding pixel intensity histograms and cell coordinate predictions of DL methods (trained on slabs 1–5). As it is clear from the histograms, the pixel dynamics of displayed MIPs (red vertical lines) cover very different ranges of intensities.

Here, the primary task is to analyze and predict cell coordinates from various slices of a human Broca’s area. Each slice was acquired independently and even though all underwent identical treatment (see Sect. "Human brain fluorescence imaging), they showed specific characteristics regarding luminosity, contrast and sources of noise (Fig. 1). The resilience of DL models to such changes is therefore crucial for their reliability. Slab membership is certainly not the only source of variability, but at the same time, is the only a-priori separable source. Here we therefore report results grouped by brain slab. A comparison between annotated and predicted cell coordinates grouped by cortical layer can be found in Supplementary Table S1.

In a first experiment, in order to account for possible covariate-shift effects due to the high variability of imaging across different slabs, all metrics (see Sect. "Localization metrics" for details) were estimated using a “leave-one-slab-out” form of cross-validation, where each slab was in turn left out for evaluating the predictions of a model trained on the other slabs (details on the dataset in Sect" Methods based on deep learning"). Cell localization performances for different deep learning methods are reported in Table 1. BCFind-v2 and StarDist (either with a ResNet or an UNet backbone) achieved the highest mean $F_1$ scores. CellPose, on the other hand, suffered from a low recall, a result in agreement with those reported by Oltmer et al.^8^, a study where the available CellPose model (trained on its own dataset^5^), was applied to the segmentation of Nissl stained hyppocampus pyramidal neurons. All DL methods, but in particular BCFind-v2 and StarDist (UNet), exhibited a high standard deviation in recall, due to the imaging differences of different slabs (some examples are shown in Fig. 1). Also, DL methods leaned towards underpredicting the total number of neurons (precision higher than recall).

**Table 1:** Mean and standard deviation of precision, recall and $F_1$ metrics computed on the validation-sets of a 6-fold cross-validation training procedure.

Method	Prec. (%)	Rec. (%)	$\text {F}_{1}$ (%)
BCFind-v2	81.2 ± 5.8	74.7 ± 8.9	77.4 ± 4.2
StarDist (ResNet)	85.1 ± 3.5	67.9 ± 6.2	75.3 ± 3.7
StarDist (UNet)	85.3 ± 5.7	67.4 ± 11.7	74.5 ± 6.4
CellPose	79.7 ± 3.7	32.4 ± 6.2	45.6 ± 5.9

Set splits are determined by the slab membership of the input volumes: volumes belonging to the same slab were always grouped together. Annotations for this experiment come from random volumes taken from slabs 1–6.

Manual inspection of predictions superimposed to the original 3D image revealed that some false positives and some false negatives are in fact due to annotation errors in the ground truth (Supplementary Figure S2). Thus, to estimate the amount of ground truth errors, we asked three experts to label a set of eight $180\times 180\times 180$ $\mu$m$^3$ volumes twice (each expert repeating the procedure after one week), obtaining a total of 48 sets of annotated soma coordinates. We then compared intra- and inter-annotator coherence using the $F_1$-measure (as defined in Sect. "Localization metrics"), obtaining $77.8\%\pm 5.7\%$ and $78.0\%\pm 6.1\%$, respectively (see details in Supplementary Table S3). This confirms the difficulty, even for humans, of correctly localizing neurons in these 3D images. Interestingly, these quantities are close to those obtained with BCFind-v2 and StarDist, as reported in Table 1.

In a second experiment, we evaluated the performances of DL models (trained on volumes from slabs 1–5) by comparing their predictions with annotations made for stereological estimates. These annotations were made by a different group of experts with a different software specifically designed for stereological purposes (Stereo Investigator, MBF Bioscience). The goal of this experiment was two-fold: to verify once again the generalization capability of DL models on unseen brain slabs and to check if they could also match stereological estimates. If DL models could be able to accurately predict stereological annotations, their predictions could produce near identical stereological estimates, making them a reliable substitute for manual annotations. All models showed an increased recall with respect to previous results (see Table 2), highlighting an improved capability in finding cells annotated in this way. On the other hand, while CellPose kept its precision unchanged, both BCFind-v2 and StarDist experienced an increase rate of false-positive detections. We speculate that such an inversion between precision and recall (in the previous experiment false-positive rate was always lower than false-negative rate) could be due to differences in the annotation process, conducted here with a more conservative approach compared to the more comprehensive one adopted in previous annotations. Particular considerations have to be mentioned for slab 18, the only one where both BCFind-v2 and StarDist reported a significant recall drop, particularly sharp on StarDist with UNet backbone. Here, as we can notice from the pixel intensity histogram in Fig.e 1, the imaging has very low levels of brightness and contrast which may have challenged the two models in detecting all cells. However, high overall $F_1$-scores and a more balanced trade-off between precision and recall, demonstrate the good reliability of DL models.

**Table 2:** Performance metrics of AI methods on stereological annotations, grouped by slab.

Slab n.	Tot. markers	Method	Prec. (%)	Rec. (%)	${\text {F}}_{1}$ (%)
6	379	BCFind-v2	69.0	**84.4**	75.9
		StarDist (ResNet)	74.7	79.7	**77.1**
		StarDist (UNet)	**81.5**	70.7	75.7
		CellPose	**81.5**	36.2	50.1
18	746	BCFind-v2	76.9	**68.7**	**72.6**
		StarDist (ResNet)	76.5	61.8	69.6
		StarDist (UNet)	**81.2**	39.9	53.5
		CellPose	78.9	47.0	58.9
30	626	BCFind-v2	72.0	**81.6**	76.5
		StarDist (ResNet)	73.6	**81.8**	77.5
		StarDist (UNet)	**77.0**	**81.9**	**79.4**
		CellPose	76.1	56.4	64.8
42	494	BCFind-v2	76.4	82.6	**79.4**
		StarDist (ResNet)	74.8	**84.6**	**79.4**
		StarDist (UNet)	78.0	81.2	**79.6**
		CellPose	**83.8**	47.2	60.4
Tot.	2245	BCFind-v2	73.8	**78.0**	**75.8**
		StarDist (ResNet)	75.6	75.4	**75.5**
		StarDist (UNet)	**78.9**	65.9	71.8
		CellPose	**79.3**	47.8	59.7

DL models are here trained on volumes from slabs 1–5. Bold values are the highest results per metric and considered set within a maximum distance of 0.5 point percentage to the best model.

### Large-scale inference

Enlarging the view, but decreasing the granularity of performance metrics, we looked at predictions on whole brain slabs. While presenting a visual and qualitative inspection we also compare predicted densities allowing for a more quantitative evaluation of DL-based models even on such large-scale inference. Here again (as in Table 2,) only models trained on volumes from slabs 1–5 were considered to compare the considered methods on previously unseen brain slices.

Figure 1 presents raw data images and DL model predictions, confirming and explaining some expectations given by the results of Table 2. In particular, the low contrast and brightness of slab 18 together with enhanced stripes artifact in the upper part of this slab, clearly affect the false-negative rate of StarDist and BCFind-v2, with higher impact on StarDist (UNet) predictions. This is also occurring in the upper-right part of slab 6. However, since the affected area is smaller in slab 6, the densities predicted by StarDist are still close to those of stereology, while on slab 18 are consistently lower (see Table 3). On slab 6, we also register the highest predicted densities for BCFind-v2, an expected result given the low precision metric on this slab (Table 2). CellPose low recall is also confirmed by the lowest predicted densities on almost every layer and slab. In layers 5 and 6 the mean densities of StarDist and BCFind-v2 predictions are nicely aligned with those of stereology. In these layers, DL models show even lower standard deviations compared to stereology highlighting better bias-variance trade-off, as also found in previous studies^8^. Supplementary Files featuring videos of dynamic model predictions and raw data on the entire z-axis traverse of slab 6 are available online. Predicted total counts are also presented in Supplementary Table S2.

**Table 3:** Predicted densities $\left( \frac{\# cells}{mm^3}\right)$ and volumes used for their derivation. Bold values are the DL estimated densities closest to stereology.

Layer	Slab	BCFind-v2	StarDist (ResNet)	StarDist (UNet)	CellPose	Stereology	Volume ($mm^3$)
3	6	16 299.80	13 826.54	**11 435.43**	6235.90	10 646.75	84.9555
	18	14 413.17	**13 245.13**	9713.07	9866.65	13 412.64	113.2260
	30	13 656.05	15 475.09	14 465.01	**10 517.81**	11 649.91	102.6240
	42	18 052.03	18 546.95	16 531.34	**9643.21**	12 251.03	51.2406
	mean	15 605.26	15 273.43	**13 036.21**	9065.89	11 990.08	
	(st. dev.)	(1709.50)	(2377.96)	(3496.18)	(1922.80)	(1156.44)	
5	6	18 017.30	14 864.79	**11 970.96**	6380.82	12 162.03	47.1015
	18	**15 780.55**	13 827.55	9843.87	9875.26	17 037.78	62.9554
	30	14 428.27	**16 011.94**	14 808.08	10 118.20	16 708.95	56.7181
	42	**20 371.37**	20 665.24	18 194.80	9541.95	20 484.54	27.4817
	mean	17 149.37	**16 342.38**	13 704.43	8979.06	16 598.32	
	(st. dev.)	(2259.02)	(3016.84)	(3485.60)	(1748.19)	(3415.10)	
6	6	15 839.03	**13 978.84**	11 517.38	7664.54	13 890.03	58.6068
	18	**13 480.22**	11 848.20	8439.84	10 217.72	17 602.81	79.6373
	30	13 316.53	**14 374.16**	13 235.04	10 340.26	15 462.57	59.0898
	42	17 273.42	**17 402.98**	15 689.57	10 576.47	23 231.62	35.0406
	mean	**14 977.30**	14 401.05	12 220.46	9699.75	17 546.76	
	(st. dev.)	(1659.38)	(2288.20)	(3074.82)	(1364.95)	(4083.97)	

**Figure 2:**
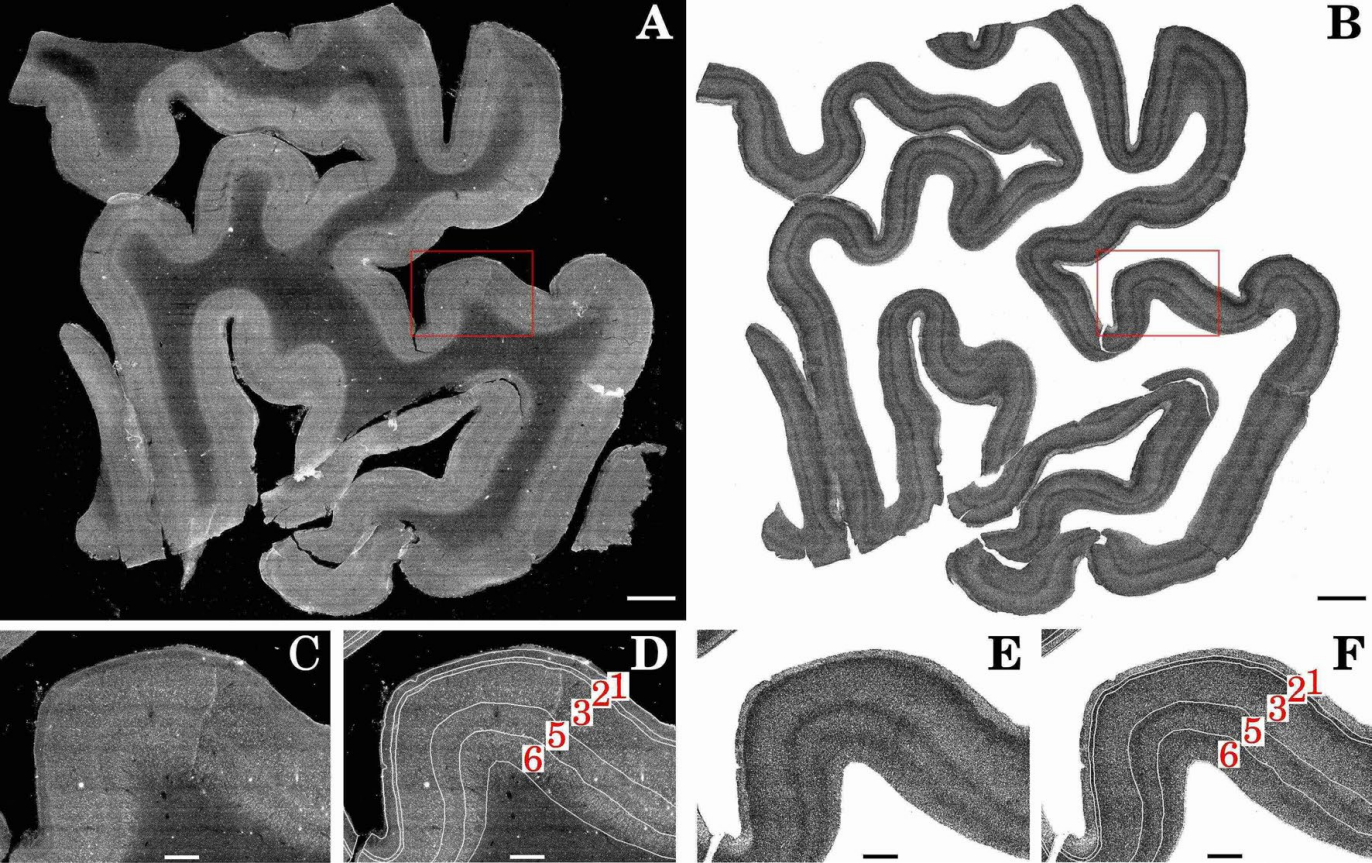
DL predictions identify cell density changes between cortical layers. (**A**) Maximum intensity projection of slab 30. (**B**) Corresponding BCFind-v2 predictions. The highlighted region of interest (ROI) (**C**) without and (**D**) with layer contours on the raw data maximum intensity projections. The same ROI on the BCFind-v2 predictions (**E**) without and (**F**) with layer contours. Red numbers in panels D and F identify the cortical layers. Scale bars: 3 mm (A, B), 750 $\mu$m (**C**–**F**).

Moreover, the point clouds obtained from DL predictions are coherent with the expected organization of neurons in the cortical layers. Indeed, all five layers of Broca’s area are easily identifiable as in the classical Nissl staining used for histological evaluation (Fig. 2). Layer 2 shows higher cell density compared to layer 1 and 3, while a dense band of cells underlines the interface between layer 3 and 5. These cell clusters are very small pyramidal cells (spiny stellate) that in most of the neocortex form a fully defined layer 4 (internal granular). In motor cortical regions they tend to cluster at the interface of layers 3 and 5, without defining a clear layer 4, but underlying the border between these two layers where groups of large pyramidal cells are very distinct^27^.

### Runtime comparisons

**Figure 3:**
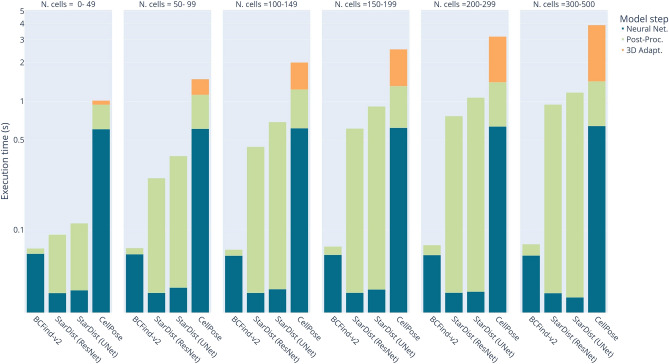
Execution times rescaled by resource percentage usage for different numbers of predicted cells. Y-axis is in log scale for better visualization. Predictions are made on 8000 volumes of $360\times 360\times 180\text { }\mu \text {m}^3$. All operations were performed on a system with an Nvidia GeForce RTX 2080 Ti, 8-cores Intel Xeon W-2123 and 128 GB RAM.

In large-scale applications, such as biological studies involving the analysis of images from several specimens, the inference time becomes a pivotal factor in determining the practicality and applicability of a model. In the following, we compare the wallclock time required by different DL-based methods to predict center coordinates in $360\times 360\times 180\text { }\mu \text {m}^3$ ($100\times 100\times 50$ voxels) volumes. Since time is highly affected by the number of predicted cells, in Fig. 3 we report the average results after binning the number of predicted cells in a sample of 8000 volumes. Moreover, to minimize the hardware and implementation impact, we rescaled the recorded times by resource percentage usage: 60% of the GPU for CellPose neural network and post-processing, 10% of the CPU for CellPose 3D adaptation, 80% of the GPU for BCFind-v2 neural network and post-processing, 10% of the GPU for StarDist neural network and 90% of the CPU for StarDist post-processing. Percentage usage and runtime were measured on a system with an Nvidia GeForce RTX 2080 Ti, 8-cores Intel Xeon W-2123 and 128 GB RAM. Figure 3 reports the execution times rescaled by the percentages mentioned above.

It is worth noting that CellPose is a 2D model, therefore 2D slices of the input volume are processed and subsequently merged to obtain 3D predictions (see Sect. "Methods based on deep learning" for details). The official implementation does not allow for predictions on user-defined batches (only large 2D images are internally tiled and batched), hence each z-plane needs to be predicted sequentially, making 3D inference particularly slow. BCFind-v2 and StarDist on the contrary, being 3D models, have much faster neural network predictions. In particular, the low-weighted StarDist neural networks (400K parameters for the ResNet and 1.2M parameters for the UNet) are faster than the BCFind-v2 neural network (18M parameters). However, the CPU-implemented post-processing of StarDist greatly increases the prediction time, while the low-weighted GPU-implemented blob detector of BCFind-v2 maintains strong speed performances even with a high number of detected cells (Fig. 3). Overall, considering the rescaled times, BCFind-v2 employed 10 min to analyze the 8000 considered volumes, StarDist (ResNet) 1 h 13 min, StarDist (UNet) 1 h 36 min and CellPose 4 h 10 min. In practice, BCFind-v2 employed an average time of 36 min to predict a whole brain slice of $4\times 4\times 0.045\text { cm}^3$, and therefore we estimate at around 30 h the time needed by BCFind-v2 to analyze a whole Broca’s area on a single GPU system, encompassing a volume of $4\times 4\times 2\text { cm}^3$ cut in 50 slices of 400 $\mu$m of thickness. Adding 60 h for ground truth generation (for training set only, see Sect. "Manual annotation effort") and training time (3 h) we can estimate a total of 93 h to apply our method to this particular region. Compared to 7 weeks of tissue preparation and imaging, this time is negligible, considering also that slab predictions can be easily parallelized if more than one GPU is available.

### Manual annotation effort

Methods for bioimage analyses can mainly extract three types of information: complete segmentation, centroid location and density or counts of objects of interest, where complete segmentation would be the finest information that could be extracted and density the coarsest. Supervised methods, as those described in this work, need human annotations of the same kind as the requested type of information. Complete manual segmentation of 3D objects is a complex and labor-intensive task, since a high number of irregular polyhedrons have to be drawn. On the other hand, having to locate object centers only is a much faster process, requiring the annotator just one point per object. Density/counts predictions also need to rely on some coordinate annotations, but as they only require a large-context understanding of the scene, they require a smaller number of manual annotations. Only 2245 cell markers were indeed needed for 3D stereology to estimate layer densities in the Broca’s area, requiring an approximate labor time of 4 working days to correctly mark the cells and segment the layers in the 4 considered brain slices. Conversely, DL models, to learn how to detect cells in 3D images, needed 22 596 ground truth markers (10 times the number of stereological annotations), requiring an approximate labor time of 81 h (16 working days, considering 5 h per day: 4 times the time needed for stereology). For what concern object segmentation, we cannot estimate the time that would be needed to segment at least the same amount of cells as for localization purposes, but it would be very considerable and inefficient.

## Discussion

Classical histological evaluation of thin sections offers the possibility to examine laminar architecture of the human cerebral cortex at high resolution; however, it is time-consuming and suffers of 2D analysis drawbacks such as poor reliability and sensitivity due to sparse observation, and sampling bias: only regions with sufficiently optimal cutting planes can be reliably analyzed. Alternatively, non-invasive neuroimaging approaches, such as MRI, allow for 3D whole-brain analyses but with low resolution and without specific cell identification. Nowadays, advances in fluorescence imaging and staining enable to reconstruct volumetric samples at high resolution and specificity. 3D reconstructions of cm-sized samples at $\mu$m-resolution produce a massive amount of data that needs to be analyzed automatically. DL approaches offer a solution to obtain a reliable cell quantification of these data. Deep models are trained discriminatively to distinguish somata from other bright elements in the image, and are therefore robust to inhomogeneous staining or to artifacts due to autofluorescence. Here, we demonstrate that BCFind-v2 and StarDist are robust tools for accurate cell coordinate predictions from volumetric samples acquired with LSFM. The simple design of BCFind-v2, specifically conceived for cell detection, significantly reduces the inference time compared to models designed for cell segmentation tasks. In this sense, BCFind-v2 enables the automatic analysis of TB-size data within the same time frame of the acquisition. The computational effort needed to analyze large image datasets is a fundamental aspect of any suitable solution for human brain research, considering the much larger size of a human brain ($\sim 1400cm^3$) compared to the mouse brain ($\sim 1~\text {cm}^3$), and the time required to process such data.

### Importance of neuron localization

Using the cell clouds produced by DL models, we could easily identify the cortical layers, demonstrating that 3D analysis can be used as a valuable solution to perform layer segmentation. Importantly, the laminar architecture changes across 3D cortical patterns (Supplementary Figure S4 shows the undulatory characteristic of acquired slabs), therefore, having a tool that maps in 3D the exact position of each cell enables the extraction of exact boundaries avoiding geometrical bias generated by the single perspective of 2D evaluation. We are therefore planning to deploy DL predictions also to segment cortical layers or subcortical nuclear structures automatically as a valuable tool to identify and analyze the cellular structure of high-resolution 3D human brain reconstructions.

Moreover, DL approaches will enable large-scale spatial analyses of molecularly and morphologically defined classes of excitatory and inhibitory neurons. Such information will provide major insight of the regional, laminar, or nuclear global distribution of specific neuronal types, not solely in the cerebral cortex, but across the neuraxis, their population numbers, and the relationships among cell types, as well as knowledge on the cellular organization of neural networks. For example previous research has identified general connectivity patterns for pyramidal neurons revealing differential, region- and layer-specific distribution of intra- and extra-cortically projecting neurons in non-human primates^27–30^. Importantly while a molecular quantitative mapping enables the definition and localization of generic classes of neurons, it can also in the context of human neuroscience, identify neuronal groups known to be differentially affected in many neuropsychiatric conditions. For example, a neurochemically identifiable subset of neocortical pyramidal neurons that provides highly specific cortico-cortical association pathways particularly affected in the course of Alzheimer’s disease, whereas GABAergic interneuron classes are generally spared^31–33^. In other conditions such as schizophrenia or autism spectrum disorder these neuronal populations present other vulnerability profiles^34–37^. In this context, a quantitative database of morpho-functional neuronal types in the human brain represents a crucial normative resource for the study of cellular changes in brain disorders. The use of automated machine learning-based quantitative approaches described in our study will be crucial to analyze differential neuronal vulnerability in brain diseases with high accuracy and sufficient throughput to generate large-scale outcomes that are not attainable with classical, manual approaches.

### Human effort aspects

In this study we have exploited annotations limited to each neuron’s soma center. This is much faster to obtain compared to whole segmentation masks. Still, the annotation effort to mark 17 000 neurons that we used as ground truth for training is considerable. Stereology has certainly a lower cost, but only count estimates are retrieved on *a priori* segmented layers by in fact analyzing a small portion of the whole reconstructed tissue. However, if there is uncertainty on the ROI, as in the case of localized brain damages, DL models could give you more fine-grained information with no spatial constraints (counts can be extracted *a posteriori* on any ROI) with the additional advantage of being completely data-driven in any portion of the tissue.

Reducing human effort involved in annotating images when several tissues are involved (as in a treatment vs. control study) is to date still an open problem that will be addressed in future research. Self-supervised training procedures^38–41^ or generative models^42–44^ are demonstrating high capabilities of learning useful features that can be later tuned on few labels to obtain accurate models. Also, models trained on data from just one brain might be adjusted to make accurate predictions on new brains by using domain adaptation algorithms^45^.

From the perspective of usage, all the analyzed DL tools offer some high-level interfaces to end-users that do not require coding skills. CellPose offers both a command-line and a graphical user interface to run various pre-trained models and possibly fine-tune a model within a human-in-the-loop approach. StarDist offers Fiji, Napari, QuPath, Icy and KNIME plugins, that however only work in inference mode without prediction corrections or fine-tuning possibilities, while training requires some Python coding skill. BCFind-v2 offers a suite of easy-to-use command-line tools for training and for basic inference modes, while Python APIs are provided for more advanced usages, as documented in the open-source code repository.

### Comparison of deep learning methods

**Figure 4:**
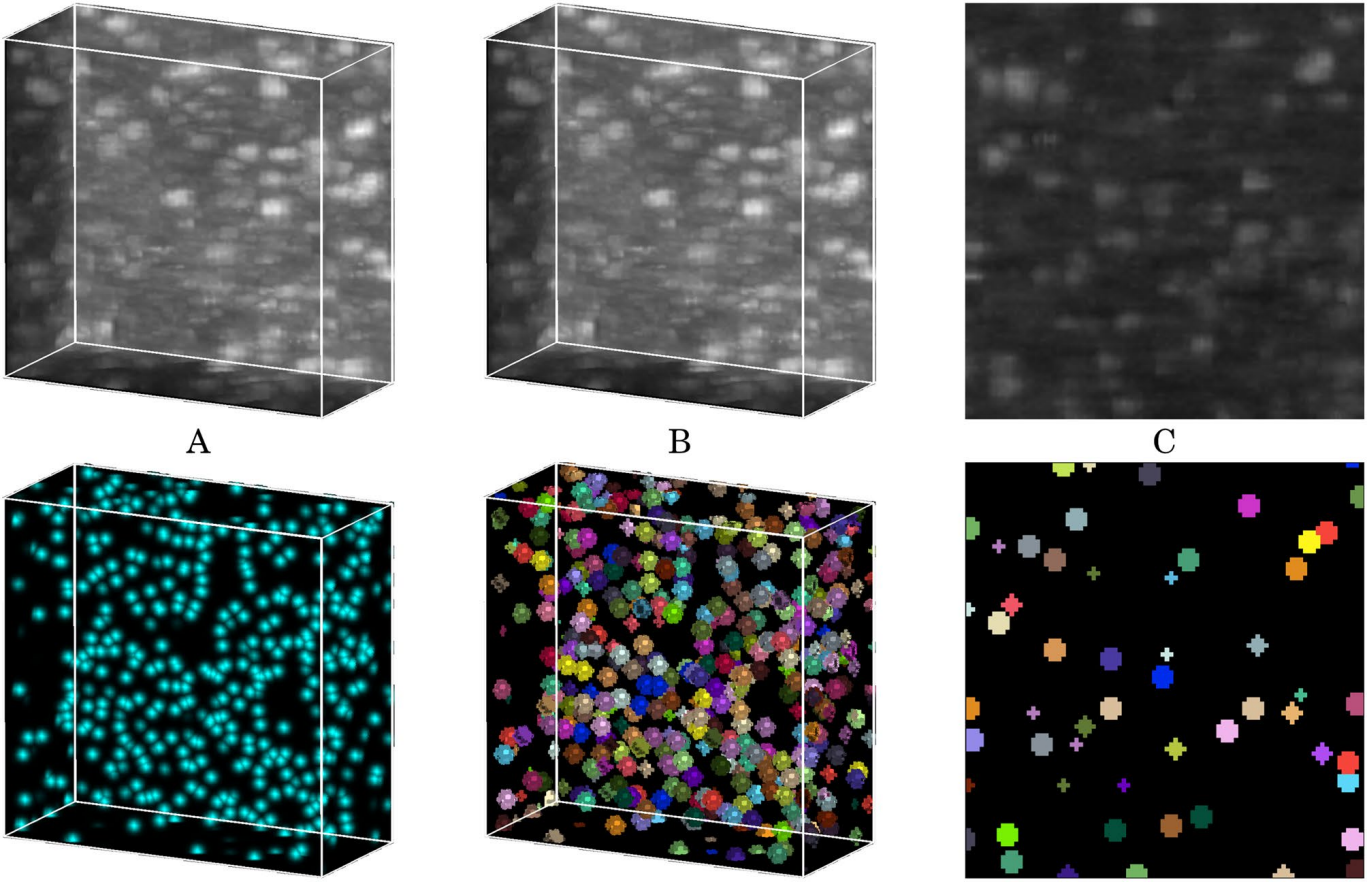
Examples of input (first row) target (second row) pairs adopted in (**A**) BCFind-v2, (**B**) StarDist and (**C**) CellPose training.

This work compares three DL methods on a large-scale 3D LSFM data set whose cells have no clear boundaries (see Fig. 4) and where the voxel-level targets were generated from point-wise annotations. BCFind was developed with this type of data in mind, is specialized for cell localization, and performs better than other approaches. However, on the one hand, CellPose is advertised as a “generalist algorithm,” and have also been tested on fluorescence microscopy images. Still, it required some effort to make it work on our data, as running it in its default configuration produced significantly worse results in terms of $F_1$ measure. Even after configuration tuning on our data (cell diameter, threshold value for hard mask generation, depth of maximum intensity projections, learning-rate, number of epochs and weight decay), its $F_1$ measure remained significantly below those of BCFind-v2 and StarDist. CellPose also turned out to be slower, mainly because its deep network operates in 2D (repeated 2D convolutions are slower than a single 3D convolution, see the comparison of neural network running time in Fig. 3, and additional computation is required for its 3D extension). On the other hand, StarDist, like BCFind, was developed for fluorescence microscopy images and it thus turned out to be much easier to adapt to our data. Indeed, differences in terms of prediction quality ($F_1$ measure) are relatively small. From the perspective of a final user, the major difference between the two methods is inference time, largely in favor of BCFind-v2 (as shown in Fig. 3). In facts, although the deep network inference time in BCFind-v2 is higher than in StarDist, the post-processing module of BCFind-v2 (based on a GPU difference-of-Gaussians implementation) takes significantly less time than finding star-convex polyhedra in StarDist. We remark that BCFind is not meant to be a general-purpose method, it focuses on predicting neuron locations (and cannot predict shapes) and its fast post-processing module might not work very well on tissues with densely packed cell, a scenario where StarDist excels.

## Methods

This section provides methodological details on Broca’s area imaging, 3D stereology and implementations of the DL models adopted in this comparative study.

The human brain tissue sample used in this study was collected by the Department of Neuropathology at the Massachusetts General Hospital (MGH) Autopsy Service (Boston, USA). Written consent was obtained from the participant prior to death, following institutional review board-approved tissue collection protocols authorized by Partners Institutional Biosafety Committee (PIBC, protocol 2003P001937). All methods were carried out in accordance with relevant guidelines and regulations. The tissue used in this project was obtained from a control subject, a 70-year-old female donor, who died of natural causes with no clinical diagnoses or neuropathology. A standard fixation protocol was used: the sample was immersed in 10% formalin for a minimum of 90 days. The sample was dissected accordingly to the Brodmann topological map^46,47^ and Broca’s area (Brodmann areas 44/45) was extracted and packed in a 2% buffered paraformaldehyde solution before performing the clearing protocol.

### Human brain fluorescence imaging

The human brain Broca’s area block was washed for one month in phosphate buffer saline solution (PBS) 0.01 M at room temperature (RT) while gently shaking. Then the human brain block was embedded with 4% agarose and cut into $400\pm 50$ $\mu$m-thick slabs with a custom-made vibratome^48^.

The slabs were treated with the SHORT^49^ protocol, a modified version of the SWITCH/TDE method^50^ that combines the SWITCH technique^51^ with the TDE clearing^52^ allowing homogenous clearing and labeling of volumetric human brain tissues. Following the SHORT protocol, the sample was first incubated in a SWITCH-off solution, consisting of 50% phosphate- buffered saline (PBS) titrated to pH 3 using HCl, 25% 0.1 M HCl, 25% 0.1 M potassium hydrogen phthalate, and 4% glutaraldehyde. The solution was replaced with PBS pH 7.4 with 1% glutaraldehyde after 24 h. The samples were washed 3 times for 2 h each in PBS at room temperature (RT) and then inactivated by overnight incubation in a solution consisting of 4% glycine and 4% acetamide at 37°C. After, samples were washed in PBS 3 times for 2 h at RT. Lipids were removed with an incubation in a solution containing 200 mM SDS, 10 mM lithium hydroxide, 40 mM boric acid for 4 days at 55°C. After this process, the samples were washed again 3 times in PBS + 0.1% Triton X-100 (PBST) at 37°C for 24 h. To lower the autofluorescence contributions the SHORT protocol has a dedicated bleaching step. Hydrogen peroxide (30% v/v) for 1 h at RT was applied. The samples were washed three times in PBS each 1 h at RT and antigen retrieval was performed using pre-heated Tris-EDTA buffer (10 mM Tris base (v/v), 1 mM EDTA solution (w/v), 0.05% Tween 20 (v/v), pH 9) for 10 min at 95°C. After cooling down to RT, the specimens were washed in DI water for 5 min each and then equilibrated with PBS for 1 h. To specifically stain the neurons, immunofluorescence was performed by incubating the sample with primary antibodies against NeuN (Merck ABN91 chicken, RRID AB_11205760) at 37°C for 7 days in PBS + 0.1% Triton (PBST) with a dilution of 1:100. Following 3 washes in PBST each of 30 min at 37°C, the samples were incubated for 5 days at 37°C with the secondary antibodies conjugated with different Alexa Fluor dyes with a dilution of 1:200 (goat anti-chicken IgY H &L Alexa Fluor$^\circledR$ 647 Abcam: ab150171) then, washed 3 times for 1 h each at 37°C. This specific staining is found particularly useful since it allows for image acquisition in the spectrum range where autofluorescence is low. To perform the 3D imaging, we mounted the samples on a glass sample holder with a 250 $\mu$m-thin quartz coverslip^53^ and placed it in an LSFM chamber filled with glycerol (91%) and distilled water for refractive index matching at 1.46. The imaging was performed with a custom-made inverted LSFM setting^12^ equipped with two orthogonal $12\times$ objectives from LaVision Biotec LVMI (Fluor $12\times$ PLAN with $12\times$ magnification, NA 0.53, WD 8.5-11 mm). The two objectives illuminate and acquire the emitted fluorescence alternately, allowing the simultaneous acquisition of two channels at an isotropic resolution of 3.6 $\mu$m after postprocessing at a volumetric speed of 0.16 cm$^3$/h. The setup is equipped with four laser sources (Cobolt: 405 nm, 488 nm, 561 nm, and 638 nm) and the fluorescence is collected through a multi-band dichroic beam splitter (Semrock Di03-R405/488/561/635-t3-55x75), before being acquired on a sCMOS camera (Hamamatsu OrcaFlash4.0 v3). Finally, the images obtained from the microscope were fused using a custom software tool written in Python called ZetaStitcher (https://github.com/lens-biophotonics/ZetaStitcher/) that was specifically developed to handle the large volumetric data produced in light-sheet microscopy. Before stitching, each 3D tile also undergoes a “deskewing” step—consisting in an affine transform with zoom, shear and rotation—to transform the images from the objective reference frame to the sample reference frame. This step is necessary to compensate for the fact that during the acquisition the sample is moved horizontally while the microscope objectives are tilted at 45°, resulting in a distortion.

### Stereology

Standard stereology provides a set of simple rules and formulas to count objects within a biological tissue acquisition with precision, accuracy, and a design free of bias caused by sampling and geometry of the objects analyzed^1,54^. In particular, it is possible to estimate parameters such as number, density, volume, surface area, or length using the systematic sampling of a region of interest (e.g., a layer of a cytoarchitecturally defined region of cortex, defined as a “volume of reference”), with an observer-independent random design. This approach gives each object under study (e.g., a neuronal population identified by a specific protein marker) the same probability to be sampled once and only once in its volume of reference, based on strict sampling criteria that are kept constant for a given object throughout the analysis^1,32,54,55^. Here, stereological analysis was performed on $400\pm 50$ $\mu$m-thick slices of Broca’s area previously cleared with SHORT^49^ and stained with immunofluorescence against NeuN for all neuronal labeling, imaged with LSFM at $3.6\times 3.6\times 3.6$ $\mu$m$^3$ voxel dimensions. The MBF Bioscience Stereo Investigator Cleared Tissue software (version 2020.1.1) with an Optical Fractionator design^1^ was used with a 3D design specifically developed for thick tissue slices fluorescently labeled as already described in Costantini et al.^12^. Briefly, for slices 6, 18, 30, and 42 of Broca’s area specimen I48, layers 3, 5 and 6 were manually outlined and their boundaries were used to estimate laminar surface areas and volume, as well as to define the reference volume of each sampling scheme. The counting frame size was $100\times 100$ $\mu$m$^2$, the grid size was $2500\times 2500$ $\mu \text {m}^2$, and the disector height was 50 $\mu$m for all sections of tissue examined generating 383 sampling sites. There was one virtual 50 $\mu$m-thick optical section for each tissue slice and the layers were contoured at the top of each sub-slab, at a $100\%$ zoom. Markers were placed at the approximate center of each sampled cell, as it came into focus within the depth of the disector. The coefficients of error of the estimates were obtained as previously described^1,32,55^ and were less than $2\%$. All Broca’s area regional and laminar boundaries were ascertained based on well-described cytoarchitectural patterns and verified in each tissue slab in which quantifications were conducted^15,16,55^.

### Methods based on deep learning

We provide here a concise descriptions of each DL approach, their implementation, and the adopted training procedure. To assess their generalization capability and ease of use, we aimed to retain the original implementations as much as possible, avoiding extensive architectural modifications that would go beyond the scope of this study. Most of the changes made primarily involve data and label preparation, which were necessary to accommodate for the specific nature of each model. Other hyper-parameters were tuned by software-specific tools provided by each library on a validation set. Training volumes were derived from six distinct brain slabs (1–6), with a total of 22 596 annotated cell coordinates distributed across 54 volumes of shape $360\times 360\times 180\text { }\mu \text {m}^3$ (see Supplementary Figure S1 for more details on the data composition). Three experts annotated these volumes with the help of the Vaa3D software^56–58^. Experimental results reported in Sect. "Cell localization" use a leave-one-slab-out cross-validation procedure where one slab in turn is left out as the test set, while the other five slabs are used for training (four slabs) and hyperparameter tuning (one slab). It is essential to note that due to the unavailability of pixel-wise cell segmentation, a more suitable type of labels for training CellPose and StarDist models, we chose not to assess the methods based on this aspect. Instead, our primary focus was solely on cell localization performances since, in this context, utilizing cell centroid labels proved to be a much more efficient and faster alternative for generating larger dataset with relative ease.

#### BCFind-v2

BCFind was introduced in the context of Purkinje cell localization in a whole mouse cerebellum^3^. It is based on a cascade of two modules: a deep learning segmentation model that aims to spot soma, and a mean-shift based blob detector that distills the coordinates of somata from the segmented image. The segmentation is *soft*: rather than asking to precisely determine membrane voxels, the deep learning model is trained using a Gaussian sphere as target. This design choice is especially tuned to the specific characteristic of 3D light sheet microscopy on cleared and marked tissue, that cannot reveal membranes. The use of soft-segmentation masks as targets for the neural network sets BCFind apart from the conventional hard-masks employed by StarDist and CellPose. The improved version used in this paper, BCFind-v2, employs a 3D UNet for segmentation^59^ and the blob detector is based on difference of Gaussian (DoG) kernels^60^. The width ($\sigma$ parameter) of each Gaussian segmentation mask is computed to avoid overlaps and thus depends on the distance to the closest cell and bounded in the interval (1, 3.5). Widths may be rescaled along *x*, *y*, *z* axes to accommodate for anisotropy. An example of input-target pair can be seen in Fig. 4A.

The UNet used in this study is trained to minimize the binary cross-entropy between predictions and normalized soft-masks. It has four encoding and four decoding blocks, each consisting of three residual blocks^61^ with full pre-activation^62^. All activations are Rectified Linear-Units (ReLU) except for the last layer which uses sigmoid activation to accommodate for the binary cross-entropy loss. As in standard 3D UNet^63^, encoder blocks reduce the size of the feature maps using max-pooling, while decoder blocks employ transposed convolutions to increase it. Inputs undergo an initial convolutional operation with $7\times 7\times 7$ kernels and are subsequently fed to a single residual block before being passed to the first block of the encoder. Notably, all residual blocks have a kernel size of $3\times 3\times 3$. The number of filters starts at 16 and increases exponentially with each successive encoder block. The neural network has been trained for 2000 epochs using stochastic gradient descent and cosine decay with warm restarts^64^ as scheduler for the learning rate.

Parameters of the DoG blob detector, as the ratio between the two Gaussian kernels, the actual sizes of the kernels, the threshold below which detected centers are removed and the minimum distance below which too close centers are merged into a single one, were tuned with tree-structured Parzen estimator (TPE) algorithm^65^, using in this case the $F_1$-score (see Sect. "Localization metrics") on validation volumes as objective function. Supplementary Figure S5 shows the whole pipeline of this method.

The code is available at https://codeberg.org/curzio/BCFind-v2.git (Sect. "Code availability").

#### StarDist

This algorithm, first presented by Schmidt et al.^66^, adopts star-convex polyhedral approximation to improve shape predictions of near-spherical objects. They show benefits in including such a-priori knowledge, especially in crowded scenes with touching cells. Their main contribution is in fact the request of radial distance predictions, in addition to class probabilities, to the neural network. Predicted radial distances to cell boundaries are used in a subsequent non-maximum suppression (NMS) step for allocating pixels to uniquely identified objects (as for instance segmentation task). Due to the 3D nature of our data, we mainly refer to the follow-up paper of StarDist^4^. In this work they extend the former 2D implementation to 3D data using a plain residual network^61^ (ResNet) with only one downsampling operator and a more efficient NMS step. Since their code offers the opportunity of using either the ResNet or the UNet as backbone architectures, we report both results.

The ResNet architecture adopted in this work comprises four residual blocks, each composed of three convolutional layers with $3\times 3\times 3$ kernel size. Similarly to BCFind-v2 and the original ResNet, inputs undergo an initial convolutional operation with $7\times 7 \times 7$ kernels. The number of filters starts at 32 and doubles every residual block. Conversely, the UNet consists of two encoder blocks and two decoder blocks, each composed of two convolutional layers with $3\times 3 \times 3$ kernel size. The number of filters starts at 32 and doubles at each encoder block. In both ResNet and UNet, since we experimented signs of overfitting, we add dropout layers at a rate of 0.3.

To satisfy StarDist need of hard instance mask labels, we generate them directly from the soft-masks adopted by BCFind-v2 thresholding them at 0.006 and then assigning unique identifiers to each object. An example of input-target pair can be seen in Fig. 4B. Training has been carried out for 1000 epochs with a learning rate of 0.001, reaching convergence and stability both on training and validation sets.

#### CellPose

CellPose^5^ has similar intuition to StarDist, but instead of adopting radial distances to approximate convex polygons, they use vertical and horizontal gradients pointing uniformly the cell centers. The UNet adopted in this work is therefore trained to predict cell probabilities and vertical and horizontal gradients. Note that no depth gradients are estimated since the model is inherently 2D. 3D data analyses is however made available by predicting on each 2D axis projection separately and then merging the three obtained results by a large agreement criterion. A modified version of the standard UNet is also presented and a large and diversified dataset is made available. Briefly, architectural modifications of the UNet involve the adoption of residual blocks^61^, addition instead of concatenation for the skip connections between encoder and decoder and the extraction of an “image-style” from the UNet bottleneck included in subsequent layers to account for between-image variability. The neural network is composed of four encoder and four decoder blocks with two residual blocks each. The residual blocks have two $3\times 3$ convolutional layers. The number of filters starts at 32 and increases exponentially every encoder block reaching a maximum of 256 filters. The network is trained to minimize the binary cross-entropy between predicted cell probabilities and hard mask labels, and to minimize the $l_2$ loss for the gradient flows.

To generate input-target pairs, we sliced the raw volumes and the targets used to train StarDist on the XY axes and adopted a maximum intensity projection (MIP) over 9 Z-planes, providing the model with some depth information and avoiding unlabeled cells due to possible misalignment between cells and hard thresholding of spherical labels. An example of input-target pair can be seen in Fig. 4C. Training has been carried out for 1000 epochs at a learning rate of 0.001. Inference is done similarly, by processing 9 z-planes MIPs with stride 1, later merged to obtain 3D predictions. Coherence between subsequent predicted masks is achieved by joining cells with an intersection-over-union (IOU) higher than 0.3. Coordinates are then extracted by taking the center of each cell mask.

### Localization metrics

Although deep models were trained to solve a 3D segmentation task (i.e., minimizing a voxel-wise loss), we are only interested in the number and the spatial location of the *centers* of the predicted cells, and the ground truth at the voxel level is artificially constructed under the assumption that all cells have approximately the same size. Hence, we do not report voxel-level metrics. Cell-level metrics were computed after matching predicted centers $\widehat{Y}=\{\widehat{y}_1,\dots ,\widehat{y}_m\}$, $\widehat{y}_i\in \mathbb {R}^3$ and ground truth centers $Y=\{y_1,\dots ,y_n\}$, $y_i\in \mathbb {R}^3$. For this purpose, we firstly constructed a bipartite graph with vertices $Y\cup \widehat{Y}$ and weights $w_{ij}=\Vert y_i-\widehat{y}_2\Vert _2$. The solution to the minimum weight bipartite matching problem^67^ on this graph is a set of prediction/ground truth pairs. A prediction $y_i$ was considered a true positive if its distance with respect to the matched ground truth center was smaller than 15 $\mu m$ and a false positive if unmatched or if matched to a ground truth center with a distance above 15 $\mu m$. A ground truth center was considered a false negative if unmatched or if its distance with respect to the matched prediction was larger than 15 $\mu m$. We then measured the quality of cell localization in terms of standard retrieval metrics: precision, $P=\frac{\textrm{TP}}{\textrm{TP} + \textrm{FP}}$, recall (or sensitivity), $R=\frac{\textrm{TP}}{\textrm{TP} + \textrm{FN}}$, and their geometric mean $F_1=2\cdot \frac{P\cdot R}{P+R}$. In general, any model that underestimates the number of cells tends to achieve high precision at the expense of recall, while any model that overestimates the number of cells tends to achieve high recall at the expense of precision. For this reason, we mainly focus on the $F_1$ measure.

Since edge effects can drastically affect model predictions at the image borders and are particularly determinant on object-wise metrics as those above, after prediction-annotation matching we removed all those matches inside a 6 (21.6 $\mu$m) pixels frame around the volume borders.

To compare DL predictions with stereological annotations we adopted particular precautions. Since stereological annotations are made such that cells overlapping the bottom-right border of the annotation box are not included, we firstly predicted on a larger volume, then we only picked predictions on a box with the same shape as annotation one but shifted top-left by 3 voxels.

### Supplementary Information

Supplementary Video 1.

Supplementary Video 2.

Supplementary Video 3.

Supplementary Video 4.

Supplementary Information 5.

## Data Availability

The samples used in this study are from the project “Imaging and Analysis Techniques to Construct a Cell Census Atlas of the Human Brain” funded by the NIH (details here: https://reporter.nih.gov/project-details/9584061). The dataset is available at this link: https://dandiarchive.org/dandiset/000026. In particular the images used in this work are from subject “I48”, NeuN staining, slices number 6, 18, 30 and 42. These files can be found under the path sub-I48/ses-SPIM/micr and they are formatted according to the Microscopy-BIDS specification^68^. Cropped volumes from these files, together with the corresponding annotated coordinates used for training DL models, can be found under the path derivatives/sub-I48/ses-SPIM/micr. Please note that this dataset, or so-called dandiset, has not been assigned a DOI yet because it is still in draft status and, at the time of writing, datasets containing OME-Zarr archives cannot be finalized (see https://github.com/dandi/handbook/blob/51114fc2411efbb3a71a3aa76029ee58dd1cbeda/docs/14_publish.md).

## Code availability

BCFind-v2 will be made public upon paper acceptance at https://codeberg.org/curzio/BCFind-v2.git. The ZetaStitcher software is freely available at https://github.com/lens-biophotonics/ZetaStitcher.
