# Supplementary material for: Deep learning-based localization algorithms on fluorescence human brain 3D reconstruction: a comparative study using stereology as a reference
Source: Sci Rep. 2024 Jun 25;14:14629. doi: 10.1038/s41598-024-65092-3 (PMC11199592; doi:10.1038/s41598-024-65092-3)
Supplement: Supplementary file 5 — Supplementary Information 5. [file 41598_2024_65092_MOESM5_ESM.pdf]

Deep Learning-based Localization Algorithms on Fluorescence Human Brain 3D Reconstruction: a Comparative Study using Stereology as a Reference

Curzio Checcucci[1,*], Bridget Wicinski[2], Giacomo Mazzamuto[3,4,6], Marina Scardigli[3,5], Josephine Ramazzotti[3], Niamh Brady[3], Francesco S. Pavone[3,4,6], Patrick R. Hof[2], Irene Costantini[3,4,7,+], and Paolo Frasconi[1,3,+]

[1]University of Florence, Department of Information Engineering, Firenze (FI), 50100, Italy
[2]Nash Family Department of Neuroscience, Friedman Brain Institute and Center for Discovery and Innovation, Icahn School of Medicine at Mount Sinai, New York (NY), 10019, USA
[3]European Laboratory for Non-Linear Spectroscopy (LENS), Sesto Fiorentino (FI), 50019, Italy
[4]National Research Council, National Institute of Optics (CNR-INO), Sesto Fiorentino (FI), 50019, Italy
[5]Current affiliation: Division of Physiology, Department of Experimental and Clinical Medicine, University of Florence, Italy
[6]University of Florence, Department of Physics, Sesto Fiorentino (FI), 50019, Italy
[7]University of Florence, Department of Biology, Sesto Fiorentino (FI), 50019, Italy
[+]These authors contributed equally to this work
[*]Corresponding author: curzio.checcucci@unifi.it

Supplementary Information

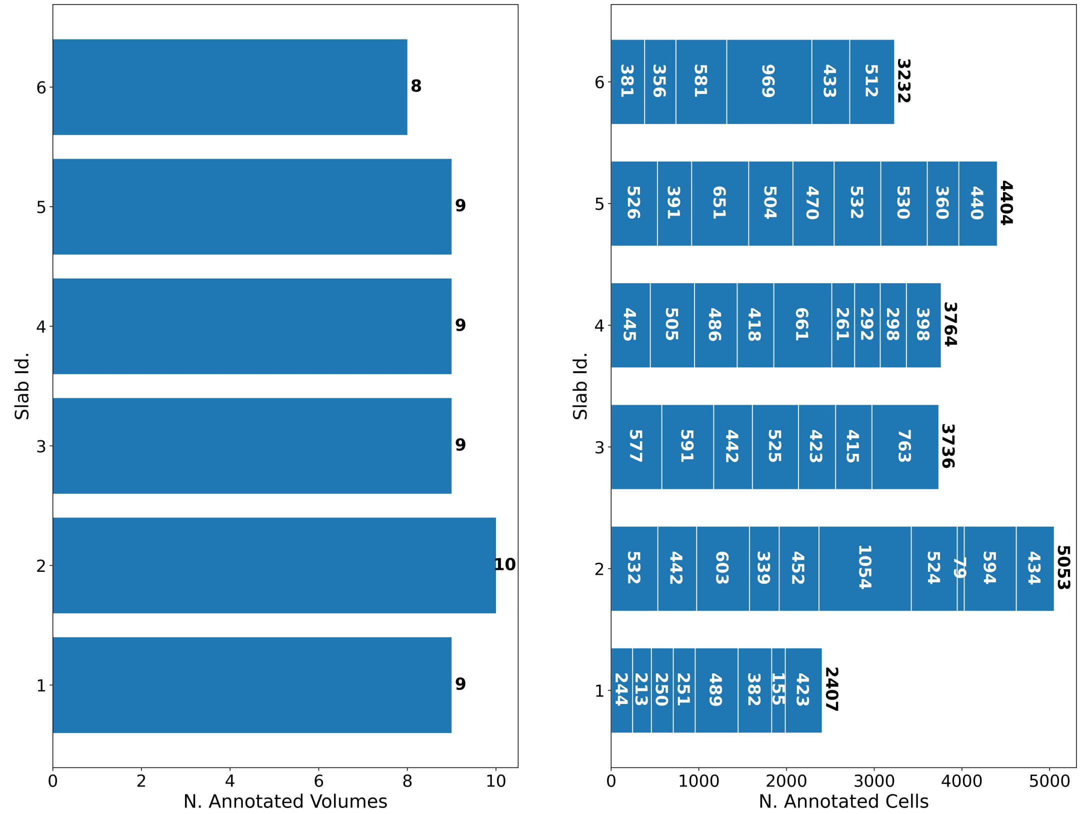

Figure S1: Distribution of annotated data. Left panel shows the number of annotated volumes per brain slab. Right panel shows the number of annotated cells per volume (white numbers) and per brain slab (black numbers). Note that some volumes do not appear in the right panel because they do not contain any annotated cell.

Table S1: Performance metrics of deep learning methods on stereological annotations grouped by layer. Bold values are the highest results per metric and considered set within a maximum distance of 0.5 point percentage to the best model.

**Layer**	**Tot. markers**	**Method**	**Prec.** (%)	**Rec.** (%)	$\mathbf{F_1}$ (%)
3	829	BCFind-v2	72.1	**81.4**	**76.5**
		StarDist (ResNet)	74.6	79.2	**76.8**
		StarDist (UNet)	78.0	69.2	73.3
		CellPose	**80.5**	48.7	59.1
5	631	BCFind-v2	73.8	**78.9**	**76.3**
		StarDist (ResNet)	74.0	74.8	74.4
		StarDist (UNet)	77.9	64.3	70.5
		CellPose	**79.4**	45.2	57.6
6	785	BCFind-v2	75.8	**73.8**	**74.8**
		StarDist (ResNet)	78.4	72.0	**75.0**
		StarDist (UNet)	**80.8**	63.7	71.2
		CellPose	78.1	51.2	61.8
Tot.	2245	BCFind-v2	73.8	**78.0**	**75.8**
		StarDist (ResNet)	75.6	75.4	**75.5**
		StarDist (UNet)	**78.9**	65.9	71.8
		CellPose	**79.3**	47.8	59.7

Table S2: Predicted counts on whole slab layers. Bold values are the DL-estimated counts closest to stereology.

Layer	Slab	BCFind-v2	Stardist (ResNet)	StarDist (UNet)	CellPose	Stereology	Volume (mm^3)
3	6	1 384 758	1 174 641	**971 503**	529 774	904 500	84.9555
	18	1 631 946	**1 499 693**	1 099 772	1 117 161	1 518 660	113.2260
	30	1 401 438	1 588 116	1 484 457	**1 079 380**	1 195 560	102.6240
	42	924 997	950 357	847 076	**494 124**	627 750	51.2406
5	6	848 642	700 154	**563 847**	300 546	572 850	47.1015
	18	**993 471**	870 519	619 725	621 701	1 072 620	62.9554
	30	818 344	**908 167**	839 886	573 885	947 700	56.7181
	42	**559 840**	567 916	500 024	262 229	562 950	27.4817
6	6	928 275	**819 255**	674 997	449 194	814 050	58.6068
	18	**1 073 528**	943 559	672 126	813 712	1 401 840	79.6373
	30	786 871	**849 366**	782 056	611 004	913 680	59.0898
	42	605 271	**609 811**	549 772	370 606	814 050	35.0406

Table S3: Intra and inter human comparisons on annotations made on 8 volumes with size 50 × 50 × 50 voxels. Three experts annotated these same volumes two times, averages over the experts and the volumes, together with the standard deviations, are reported.

	**Prec.** (%) (st. dev.)	**Rec.** (%) (st. dev.)	$\mathbf{F_1}$ (%) (st. dev.)
Intra-human	72.2 (8.9)	86.2 (10.6)	77.8 (5.7)
Inter-human	83.5 (8.9)	74.8 (10.9)	78.0 (6.1)

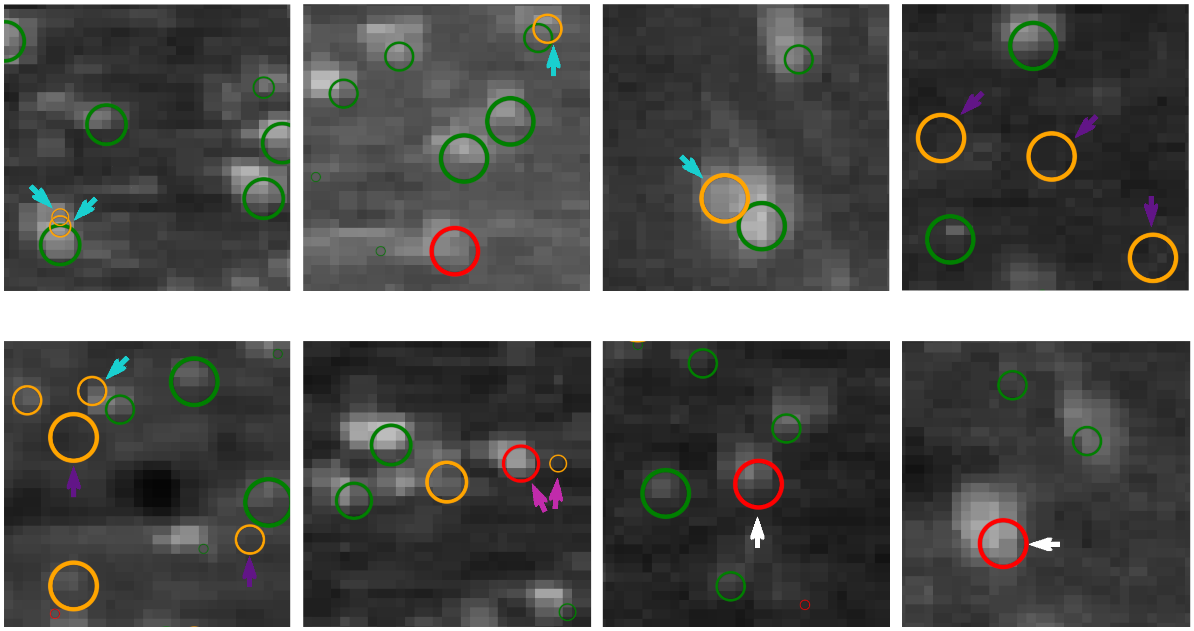

Figure S2: Ground truth mistakes and BCFind-v2 predictions. Some cells are annotated multiple times (light-blue arrows), some annotations fall into dark regions (purple arrows) or close to actual cells whose correct detection appear as false-positive (pink arrows), some false-positives correctly detect a cell (white arrows). Each image is a 126 × 126 μm^2 plane taken from a test volume (Section 2.1 of main text) overlaid with the scatter plot of BCFind-v2 predictions. Green circles denote true-positives predictions, red circles false-positives and orange circles false-negatives. Circle sizes are proportional to the proximity of predicted z-coordinates to the displayed plane and have a maximum radius of 15 μm. Best viewed in color.

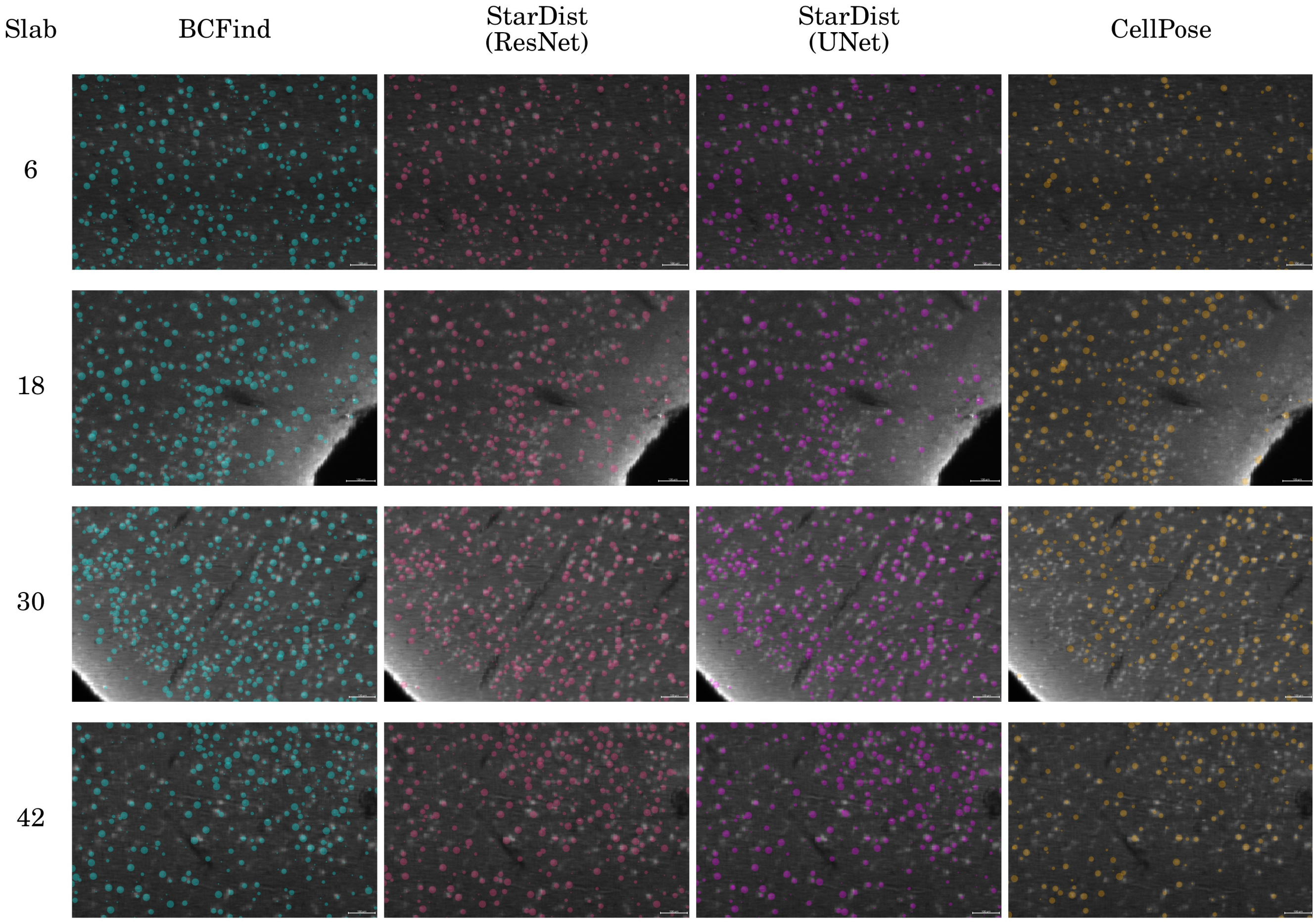

Figure S3: DL predictions on a small 2D RoI for each considered slab. Point sizes represent the proximity of predicted z-coordinates to the displayed plane with a maximum distance of 15 μm (each point radius). Scale bars are 100 μm long. Better viewed in color and zooming in.

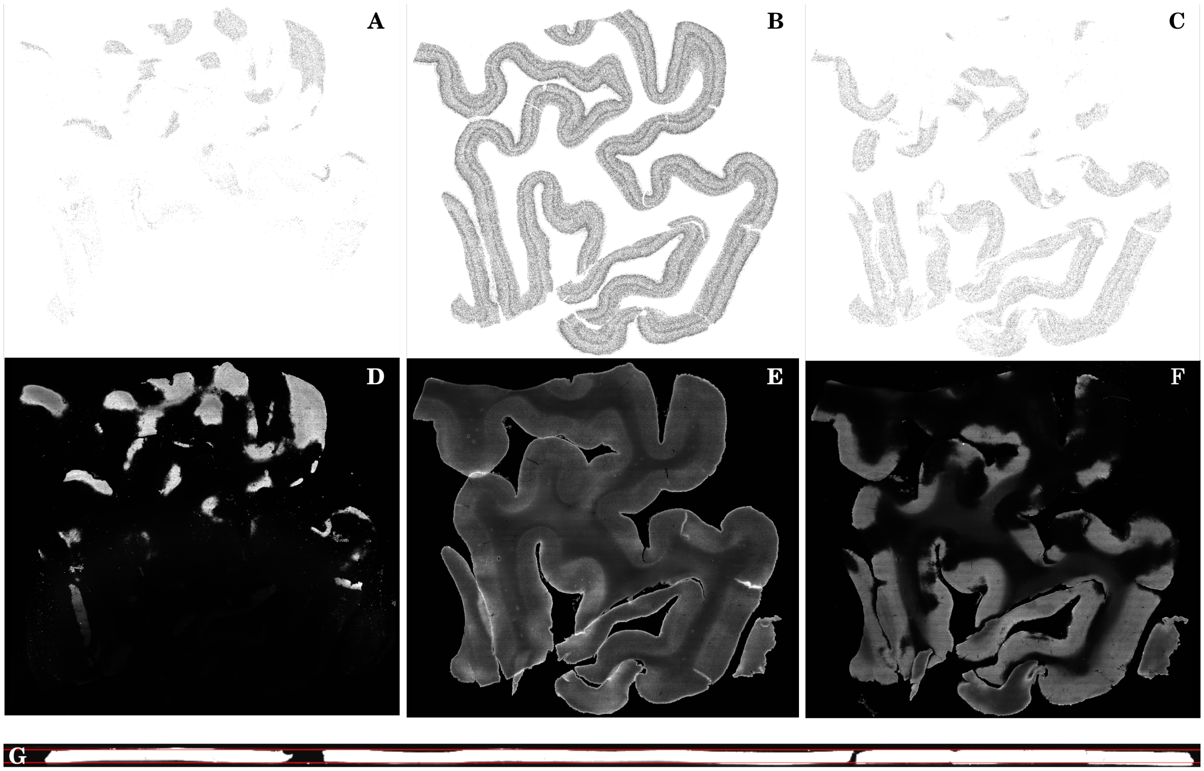

Figure S4: Undulatory feature of acquired slabs. BCFind-v2 predictions and raw data plane at (A, D) $Z = start$, (B, E) $Z = mid$ and (C, F) $Z = end$ of slab 30. (G) ZY projection of slab 30, red horizontal lines show $Z = start$ and $Z = end$ considered by the above images and stereological estimates. Panel G is intentionally highly saturated to better highlight the tissue shape.

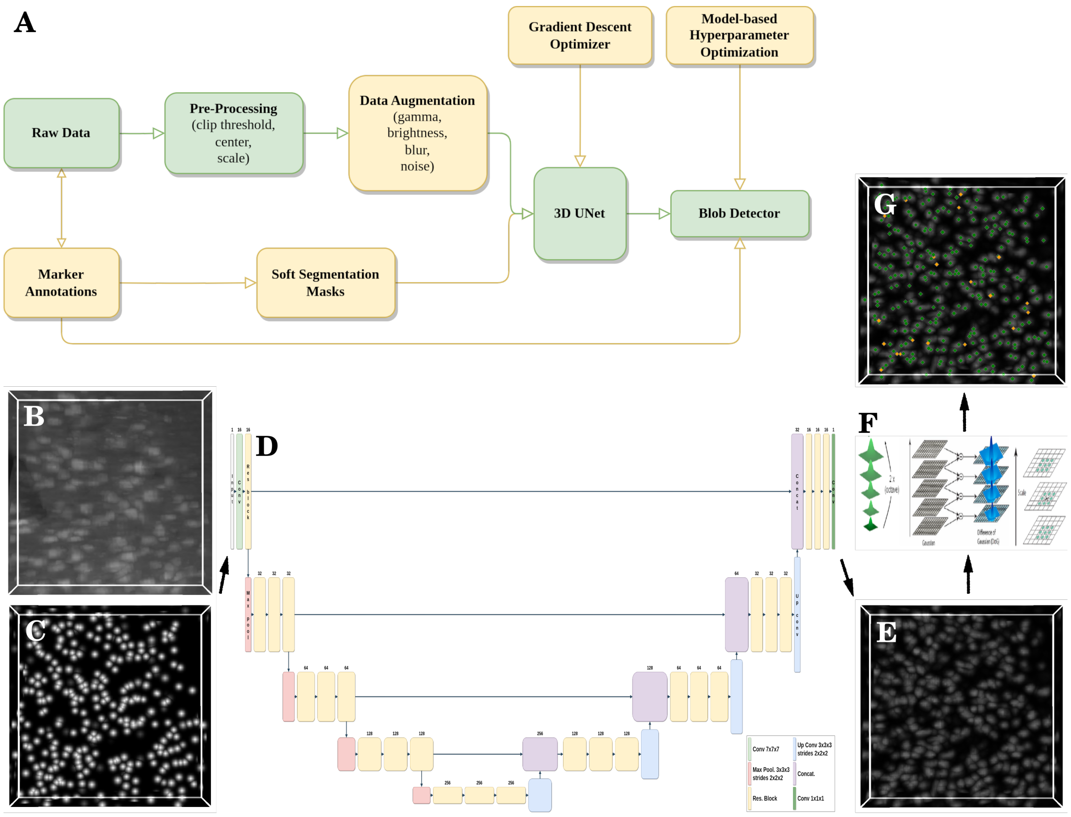

Figure S5: BCFind-v2. (A) Schematic representation of the whole pipeline. Yellow boxes and edges are only needed for training. (B) Input and (C) target pair for 3D UNet training. (D) 3D UNet architecture. (E) 3D UNet prediction. (F) Schematic representation of DoG for blob detection. (G) DoG predictions: green points are true positives, orange points false negatives and red points false positives.

Overall effort required to prepare, image, and analyze a whole Broca's area

The preparation of a ($4 \times 4 \times 2$ cm^3) tissue sample, approximately corresponding to a whole human Broca's area, takes about two weeks for tissue clearing and another two weeks for specific staining (all the slabs can be treated in parallel as described in detail by Di Meo et al. 2024 [2] and multiple staining can be performed up to 4 colors). Our custom-made LSFM has an image velocity of 47 frames/s [1], and one 4×4 cm^2 400 μm- thick slab can be imaged in approximately 3 hours. Once prepared, a whole block of $\sim$50 slabs (a Broca's area is around 2 cm thick) can be imaged at the microscope in approximately 13 working days (considering 4 slabs every day). Post-processing of the data (mainly image reslicing and stitching) requires approximately 1 h per slice. Thus, to obtain images for a 3D $4 \times 4 \times 2$ cm^3 block of Broca's area at a 3.6 μm isotropic resolution we need approximately six weeks. Creating the ground truth for supervised learning would require about 60 hours (we spent 80 hours but 25% of the annotations were used to estimate the F_1-measure on held-out data and since the method is already validated, we will not need them). Training BCFind-v2 and running inference on the whole set of slabs would take 3 and 30 hours, respectively, on a single GPU, i.e. a modest amount of the overall time (interestingly, inference with StarDist or Cellpose would take approximately an order of magnitude more, as shown in Fig.3). Hence, a whole Broca's area can be prepared, imaged, and analyzed in less than two months in an idealized full-time working regime.

References

[1] Irene Costantini et al. "A cellular resolution atlas of Broca's area". In: *Science Advances* 9.41 (2023), eadg3844.

[2] Danila Di Meo et al. "Optical Clearing and Labeling for Light-sheet Fluorescence Microscopy in Large-scale Human Brain Imaging." In: *Journal of Visualized Experiments: Jove* 203 (2024).
